# Effects of platelet‐rich fibrin on human gingival and periodontal ligament fibroblast proliferation from chronic periodontitis versus periodontally healthy subjects

**DOI:** 10.1002/cre2.370

**Published:** 2021-01-14

**Authors:** Apoorv Goel, L. Jack Windsor, Richard L. Gregory, Steven B. Blanchard, Yusuke Hamada

**Affiliations:** ^1^ Department of Periodontology Meharry Medical College School of Dentistry Nashville Tennessee USA; ^2^ Department of Biomedical Sciences and Comprehensive Care Indiana University School of Dentistry Indianapolis Indiana USA; ^3^ Department of Periodontology Indiana University School of Dentistry Indianapolis Indiana USA

**Keywords:** fibroblasts, healing, periodontitis, platelet‐rich fibrin

## Abstract

**Background:**

Platelet‐rich fibrin (PRF), an autogenous blood concentrate, contains multiple growth factors and is used as an adjunct in the periodontal regeneration and implant site development procedures to stimulate wound healing. Patient‐related factors such as chronic periodontitis may affect the quality of PRF.

**Objectives:**

This study aimed to investigate and compare PRF's effects from patients diagnosed with generalized moderate or severe chronic periodontitis to patients who presented with intact periodontium on human gingival fibroblast (HGF) and human periodontal ligament fibroblast (HPLF) proliferation.

**Materials and methods:**

A total of 33 ml of whole intravenous blood was collected from each subject and centrifuged at 2700 rpm for 12 min in three 10 ml tubes, and 3 ml of blood was used for Complete Blood Count analysis. Three PRF clots were compressed to produce the membranes and liquid exudate. PRF membrane and 10% liquid exudate were exposed to 20,000 HPLFs/well or 25,000 HGFs/well in triplets from each subject in a 48 cell well plate. After 72 h of incubation, the conditioned media were evaluated by Water Soluble Tetrazolium‐1 assays to determine fibroblast proliferation. Controls included cells alone and media without cells. Complete blood counts were measured.

**Results:**

Subjects in both groups were age and gender‐matched (intact 46.7 ± 11.4 years and periodontitis 54.8 ± 10.4 years, *p*‐value = 0.1344). Body Mass Index and White Blood Corpuscles in the periodontitis group was significantly higher than the intact group (*p* = 0.0176 and *p* = 0.0038) whereas no differences were seen for Red Blood Corpuscles (*p* = 0.2020), Hemoglobin (*p* = 0.2290) and Platelets (*p* = 4,094). There were no significant differences in the HGF and HPLF proliferation with PRF exudates and membranes between intact periodontium and periodontitis groups (all *p* > 0.05). However, PRF exudates in both groups induced significant more cell proliferation when compared to PRF membranes.

**Conclusions:**

PRF exudates induced significant proliferation of fibroblasts and can play a vital role in wound healing. The current study concluded that PRF membranes, in combination with PRF exudates, can be utilized for their therapeutic and wound healing potential, not affected by the periodontal condition of the patient.

## 1 INTRODUCTION

Platelets are components of blood‐derived from megakaryocytes and have an average lifespan of 8–12 days. Platelets play a significant role in initiating hemostasis at the site of disrupted vascular endothelium and providing a natural source of factors that help wound healing and tissue regeneration (Gawaz & Vogel, 2013). Various cytokines and growth factors are released from platelets that include platelet‐derived growth factor (PDGF), transforming growth factor‐beta 1 (TGF‐β1), vascular endothelial growth factor (VEGF), epidermal growth factor (EGF), a platelet‐derived angiogenic factor, and insulin‐like growth factor (IGF) (Gawaz & Vogel, 2013; Suarez‐Lopez Del Amo et al., 2015). During hemostasis, platelets become entrapped in the fibrin clot and release cytokines and growth factors upon degranulation. The use of fibrin clots has been investigated as therapeutic tools to aid in periodontal repair and regeneration of periodontal defects (Del Corso et al., 2012; Gawaz & Vogel, 2013; Suarez‐Lopez Del Amo et al., 2015). Two generations of platelet‐rich concentrates and their roles in healing have been widely studied (Choukroun et al., 2006; Del Corso et al., 2012; Dohan et al., 2006; Simonpieri et al., 2012). The first generation of platelet‐rich concentrates is referred to as platelet‐rich plasma (PRP) and is obtained from autologous whole venous blood. The procedure to process PRP includes citrate phosphate dextrose, which is an anticoagulant (Del Corso et al., 2012; Dohan et al., 2006; Simonpieri et al., 2012).

The second generation of platelet‐rich concentrates is referred to as platelet‐rich fibrin (PRF). The preparation of PRF involves centrifugation without the addition of anticoagulants. Early versions of PRF, also called Leukocyte‐PRF (L‐PRF), was formed by centrifugation at 2700 rpm for 12 min, while another protocol utilizes a centrifugation speed of 1500 rpm for 14 mins and is referred to as Advanced‐PRF (A‐PRF; Del Corso et al., 2012; Kobayashi et al., 2016). Due to the absence of anticoagulants, activation of platelets is initiated, which leads to the completion of the coagulation cascade (Dohan et al., 2006). Initially, fibrinogen accumulates in the upper part of the centrifuged tube and, with the effect of circulating thrombin, forms fibrin. This fibrin traps the platelets and leukocytes. The PRF's therapeutic potential is due to their release of cytokines and growth factors.

PRP, L‐PRF, and A‐PRF release growth factors such as PDGF‐AA, PDGF‐AB, PDGF‐BB, TGF‐β, VEGF, EGF, and IGF, but their release kinetics are different (Kobayashi et al., 2016). To understand PRF's role in the wound healing process or repair, it is crucial to study how the release of growth factors and cytokines from the fibrin affects cell migration, proliferation, and maturation. The periodontium, a unique complex structure of soft and hard tissues consisting of gingival connective tissue, periodontal ligament tissue, cementum, and bone, tends to repair using collagenous fibrous tissue epithelial downgrowth. Human gingival fibroblasts (HGF) and human periodontal ligament fibroblasts (HPLF) play a vital role in the maintenance of periodontal health, as well as in wound healing following injury or surgical procedures (Basdra & Komposch, 1997). Therefore, the proliferation of HGF and HPLF could aid in faster repair and regeneration of the periodontal structures.

Studies showed that PRF membranes (Vahabi et al., 2015) and fibrin clot (Fujioka‐Kobayashi et al., 2017) lead to the statistically significant proliferation of HGF at 24 h, and the same trend was noticed with increased numbers of cells at 3 and 5 days. The source of blood for these PRF was from healthy individuals (Vahabi et al., 2015). Patients with chronic inflammatory diseases (i.e., chronic periodontitis and diabetes) have increased systemic levels of pro‐inflammatory cytokines and growth factors. A recent study (Chang et al., 2019) quantified the growth factors released from PRF, serum concentrations of the cytokines (IL‐1β, IL‐6, and TNF‐α), and Complete Blood Count (CBC) analysis from periodontitis versus healthy subjects and found no significant differences between groups. The periodontitis group in the study (Chang et al., 2019) showed higher White Blood Cells (WBC) with no correlation to the growth factors released from the PRF. No study has investigated the role of PRF from patients diagnosed with chronic periodontitis on HGF and HPLF. Therefore, this study's objective was to compare the effects of autologous PRF on HGF and HPLF proliferation from subjects with periodontitis versus subjects with a healthy periodontium.

## 2 MATERIALS AND METHODS

### 2.1 Subjects

For this study, blood samples were collected from subjects recruited with approval from the Indiana University Institutional Review Board (protocol# 1707485479 and protocol# 1704165172). As this study was the continuation, inclusion, and exclusion criteria are the same as in Chang et al. (2019). The patient's criteria are described briefly here. The periodontitis group included patients with a diagnosis of generalized moderate to severe chronic periodontitis. Patients required to have minimum clinical attachment loss of 3 mm present at greater than 30% of teeth, need to be presented with bleeding on probing, probing depth ≥5 mm, and evident radiographic bone loss of a minimum of 16%. The healthy group was subjects with intact periodontium. The patients included in the study were between 30 and 65 years of age and nonsmokers with controlled or no systemic diseases. The patients excluded from the study were with uncontrolled systemic disorders. Systemic diagnosis included: diabetes (HbA1c ≥ 6.5%), immunocompromised conditions, uncontrolled hypertension, and other heart‐related diseases in the past 6 months. Other exclusion criteria were: (a) history of periodontal therapy (phase 1 or 2) within the last 2 years; (b) body mass index (BMI) of <18.5 or ≥ 40 kg/m^2^; (c) receiving anticancer therapy (chemotherapy and radiotherapy); (d) currently on medications that included: corticosteroids, anticoagulants, antiplatelets, nonsteroidal anti‐inflammatory drugs, or antibiotic treatment within last 6 months; and (e) history of drug or substance abuse.

### 2.2 PRF collection

Blood collection procedures were followed as per Chang et al. (2019). During the Chang et al. study, 33 ml of blood was collected for the current study, of which 3 ml was used for CBC analysis, and 30 ml was collected in three tubes. L‐PRF clots were produced by centrifuging the tubes at 2700 rpm which is equivalent to 650 Relative Centrifuge Force‐max (RCF) for 12 min without anticoagulants to allow for fibrin clot formation. The L‐PRF clots were produced using the Clinifuge Centrifugation Device (Kendro Laboratory Products, Hanau, Germany) using 10‐ml glass tubes. The clot was collected in the middle of the tube between the red corpuscles at the bottom and the acellular plasma at the top. The clots were then compressed in a PRF box (OSUNG MND Co, Kyonggi‐do, Korea) for 5 min to form a flattened PRF membrane. Each membrane was uniformly trimmed into three 10 × 10 mm^2^ membranes for a total of nine membrane samples from each subject. The PRF exudate (E) collected in the PRF box's lower chamber after compressing the fibrin clot was collected and frozen along with the membranes at −80°C.

### 2.3 Cell culture

HGFs were originally cultured from samples of clinically noninflamed gingival connective tissues collected for Al‐Shibani and Windsor (2008). These HGFs were frozen at −80°C after being processed as in previous studies (Al‐Shibani & Windsor, 2008; Khaled et al., 2013). HPLFs were bought commercially (Catalog No. 2630, ScienCell™ Research Laboratories, 6076 Corte Del Cedro, Carlsbad, CA 92011). Both HGFs and HPLFs were cultured in the cell culture dishes with Dulbecco's Modified Eagle's Medium (DMEM, Corning™, Mediatech, Inc., Manassas, Virginia, Catalog No. 10‐013‐CV) supplemented with 10% fetal bovine serum (FBS), 200 mM l‐glutamine, 100 U/ml penicillin, 50 μg/ml gentamycin, and 250 μg/ml amphotericin B and incubated at 37°C and 5% CO_2_. Cells at passages 3–5 were used in the experiments.

### 2.4 Measurement of cellular metabolism by water‐soluble tetrazolium‐1 (WST‐1) assay to measure cellular proliferation

Mitochondrial dehydrogenase activities were determined to utilize the WST‐1 assay (Roche Applied Science, Indianapolis, Indiana). The assay principle is based on the fact that the tetrazolium salts are cleaved to formazan by the mitochondrial dehydrogenases. An expansion in the viable cells' metabolism results in an increase in the overall activity of mitochondrial dehydrogenases in the sample. This augmentation in enzyme activity leads to an increase in the amount of formazan dye formed, which directly correlates to the metabolism of the culture's metabolically active cells. The HGFs and HPLFs were detached from cell culture dishes with 0.25% EDTA trypsin, pelleted, resuspended in fresh media, and seeded as 25,000 HGFs/well or 20,000 HPLFs/well in 48‐well plates with 1000 μl DMEM +10% FBS. The plates were then incubated for 24 h to allow the fibroblasts to attach, the media was then removed, and both fibroblast types were exposed in triplet to 10 × 10 mm^2^ PRF membranes or 10% PRF exudate liquid along with 1000 μl DMEM without fetal bovine serum. The positive control consisted of the fibroblasts (HGF and HPLF) alone in 1000 μl DMEM without fetal bovine serum, and the negative control was 1,000 μl DMEM alone. After 72 h, the media was removed from the 48‐well plates, and 200 μl of 10% WST‐1 reagent was added in each well. The plate was incubated for 1 h at 37°C and 5% CO^2^. A 100 μl sample from each well was placed in a 96‐well plate, and the absorbance of the samples against 10% WST‐1 reagent as the blank was measured using a microplate reader (Titertek, Flow laboratories, Mclean, Virginia) at 450 nm. Based on the absorbance values of each sample, the cellular proliferation percentage was calculated by comparing it with the positive control (untreated cells) by using the following equation:

$$\text{Cell Proliferation}\%.=\left(\text{experiment value}-\text{negative control}\right)/\left(\text{positive control}-\text{negative control}\right)\times 100\%$$

### 2.5 Statistical analysis

Cell types HGF and HPLF were analyzed separately. Cell proliferation data were analyzed using two‐way ANOVA to exam the effect of samples (cells, cells + E, and cells + PRF), groups (healthy and periodontitis), as well as the interactions between samples and groups. All pair‐wise comparisons from ANOVA analysis were made using Fisher's Protected Least Significant Differences to control the overall significance level at 5%. Cell proliferation was summarized by groups and samples.

Demographic characteristics (age and BMI) and lab data (WBC, RBC, Hgb, and Platelet) were summarized by groups. Two independent samples *t*‐tests were performed for continuous outcomes, and Fisher's exact tests were performed for categorical outcomes to test the difference between test and control groups.

## 3 RESULTS

### 3.1 Subject characteristics and laboratory data

Blood samples of nine subjects in each group were analyzed in this study (Table 1). Subjects in both groups were age‐matched (healthy 46.7 ± 11.4 years and periodontitis 54.8 ± 10.4 years, *p*‐value = 0.1344). Body Mass Index (BMI) and White Blood Corpuscles (WBC) in the periodontitis group was significantly higher than the healthy group (*p* = 0.0176 and *p* = 0.0038) whereas no differences were seen for Red Blood Corpuscles (RBC, *p* = 0.2020), Hemoglobin (Hgb, *p* = 0.2290) and Platelets (*p* = 4,094).

**TABLE 1:** Patient characteristics and laboratory data for subjects with healthy periodontium and periodontitis presented in mean (*SD*)

Subject characteristics	Healthy	Periodontitis	*p* Value
	Mean (*SD*)	Mean (*SD*)	
Age (years)	46.7 (± 11.4)	54.8 (± 10.4)	0.1344
BMI (kg/m^2^)	25.1 (± 2.4)	29.0 (± 3.8)*	0.0176
WBC (k/cumm)	5.1 (± 1.0)	7.5 (± 1.5)*	0.0038
RBC (million/cumm)	5.0 (± 0.5)	4.7 (± 0.3)	0.2020
Hgb (GM/dl)	14.5 (± 1.3)	13.8 (± 1.1)	0.2290
Platelet (k/cumm)	221.3 (± 50.9)	249.5 (± 73.3)	0.4094
Gender	*M* = 6, *F* = 3	*M* = 6, F = 3	—

Abbreviations: BMI, Body Mass Index; Hgb, Hemoglobin; RBC, Red Blood Corpuscles; WBC, White Blood Corpuscles.

* *p* < 0.005.

### 3.2 Cell proliferation

**FIGURE 1:**
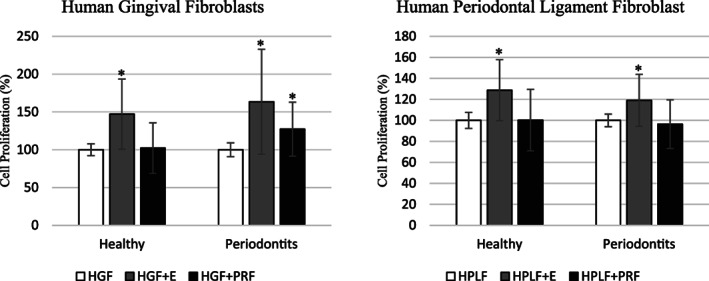
Cell proliferation comparison (within groups) is shown graphically. Cell proliferation is adjusted keeping positive control proliferation at 100%. Human Gingival Fibroblast, HPLF: Human Periodontal Ligament Fibroblast, PRF: Platelet‐Rich Fibrin, E: PRF Exudate. * denotes *p* < 0.005 when compared to control cells (HGF or HPLF) within group

**FIGURE 2:**
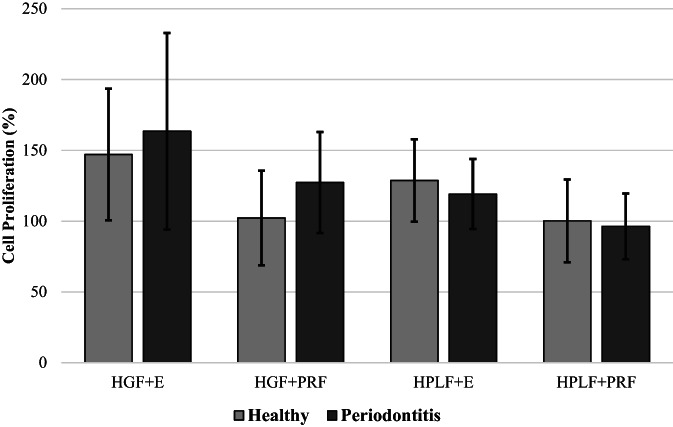
Cell proliferation comparison are shown graphically. There were no significant differences between healthy and periodontitis groups for HGF and HPLF proliferation with PRF membranes and PRF exudates. Cell proliferation is adjusted keeping positive control proliferation at 100%. E, PRF Exudate; HGF, Human Gingival Fibroblast; HPLF, Human Periodontal Ligament Fibroblast; PRF, Platelet‐Rich Fibrin

Based on the equation to calculate cell proliferation, positive controls (i.e., HGF and HPLF) proliferation were set at 100%. The mean cell proliferation for HGF or HPLF with PRF exudates or PRF membranes for healthy and periodontitis groups is described in Table 2. In the healthy group, HGF + E (147.1 ± 46.4%) was significantly higher for proliferation than HGF + PRF (102.2 ± 33.3%, *p* < 0.0001, Tables 2 and 3, Figure 1). Also, in the healthy group HPLF+E (128.7 ± 29.1%) proliferation was significantly higher than HPLF+PRF (100.2 ± 29.3%, *p* < 0.0001, Tables 2 and 4, Figure 1). In the periodontitis group, HGF + E (163.5 ± 69.4%) induced significantly higher proliferation than HGF + PRF (127.3 ± 35.7%, *p* < 0.0002, Table 2 and 3, Figure 1). Also, in the periodontitis group, HPLF+E (119.1 ± 24.7%) was significantly higher for proliferation than HPLF+PRF (96.3 ± 23.2%, *p* < 0.0001, Table 2 and 4, Figure 1). HGF + PRF in the periodontitis group was the only cell group with PRF membranes to show proliferation (127.3 ± 35.7%, Tables 3 and 4). There were no significant differences between the healthy and periodontitis groups (*p* > 0.05, Table 5 and Figure 2).

**TABLE 2:** Cell proliferation (%) summary for a different group of cells presented in mean (SD: standard deviation)

Group	Samples	Mean cell proliferation% (SD)
Healthy	HGF	100.0 (7.9)
Healthy	HGF + E	147.1 (46.4)
Healthy	HGF + PRF	102.2 (33.3)
Healthy	HPLF	100.0 (7.6)
Healthy	HPLF + E	128.7 (29.1)
Healthy	HPLF + PRF	100.2 (29.3)
Periodontitis	HGF	100.0 (9.1)
Periodontitis	HGF + E	163.5 (69.4)
Periodontitis	HGF + PRF	127.3 (35.7)
Periodontitis	HPLF	100.0 (6.0)
Periodontitis	HPLF + E	119.1 (24.7)
Periodontitis	HPLF + PRF	96.3 (23.2)

Abbreviations: E, PRF Exudate; HGF, Human Gingival Fibroblast; HPLF, Human Periodontal Ligament Fibroblast; PRF, Platelet‐Rich Fibrin.

**TABLE 3:** Cell proliferation comparison within group for Human Gingival Fibroblast (HGF)

Group	Samples	*p* Value (significance)
Healthy	HGF and HGF + PRF	0.7976
	HGF < HGF + E	<0.0001*
	HGF + E > HGF + PRF	<0.0001*
Periodontitis	HGF < HGF + E	<0.0001*
	HGF < HGF + PRF	0.0033*
	HGF + E > HGF + PRF	0.0002*

Abbreviations: E, PRF Exudate; PRF: Platelet‐Rich Fibrin.

* denotes *p* < 0.005.

**TABLE 4:** Cell proliferation comparison within group for Human Periodontal Ligament Fibroblast (HPLF)

Group	Samples	*p* Value (significance)
Healthy	HPLF and HPLF + PRF	0.9723
	HPLF < HPLF + E	<0.0001*
	HPLF + E > HPLF + PRF	<0.0001*
Periodontitis	HPLF & HPLF + PRF	0.4490
	HPLF < HPLF + E	0.0004*
	HPLF+E > HPLF + PRF	<0.0001*

Abbreviations: E, PRF Exudate; PRF: Platelet‐Rich Fibrin.

* denotes *p* < 0.005.

**TABLE 5:** Cell proliferation comparisons between healthy and periodontitis groups

Cells	Result	Controlling	*p* Value (significance)
HGF	Healthy and periodontitis	HGF + E	0.2730
	Healthy and periodontitis	HGF + PRF	0.0980
HPLF	Healthy and periodontitis	HPLF+E	0.2419
	Healthy and periodontitis	HPLF+PRF	0.6318

Abbreviations: E, PRF Exudate; HGF, Human Gingival Fibroblast; HPLF, Human Periodontal Ligament Fibroblast; PRF, Platelet‐Rich Fibrin.

## 4 DISCUSSION

The results from this study found that PRF exudates significantly induced proliferation of HGF and HPLF in both the healthy and periodontitis groups (Tables 3 and 4, and Figure 1). Whereas, only HGF + PRF in the periodontitis group showed significantly higher proliferation compare to HGF + PRF from healthy individuals (Table 3 and Figure 1). No HPLF proliferation was seen with PRF membranes in healthy and periodontitis groups (Tables 3 and 4). Also, no significant differences in cell proliferation were noticed with PRF exudates and membranes between the healthy and periodontitis group. This suggests that cell proliferation studied for healthy and periodontitis groups were not affected by the patient's periodontal status.

A recent study by Chang et al. (2019) measured the amount of certain growth factors (i.e., EGF, IGF‐1, PDGF‐BB, TGF‐β1, and VEGF) from PRF membranes and exudates from healthy and periodontitis subjects and found no significant differences between the groups (all *p* > 0.05). Chang et al. concluded that both PRF membranes and exudates contained growth factors. The concentration of the growth factors in the PRF membranes was much higher when compared to the PRF exudates (Chang et al., 2019). Chang's study showed that higher amounts of PDGF‐BB and TGF‐β1 were released in the initial 24 h from the PRF membranes of the periodontitis group and may have played a role in inducing HGF proliferation. HGF or HPLF proliferation due to PRF exudates could be due to growth factors that were not evaluated in the study by Chang et al. and further studies are required to analyze the PRF exudates. The current study agrees with Chang et al. that PRF exudate can be utilized for hydrating graft materials and can be used in combination with PRF membranes to maximize a fibrin clot's potential.

The current study found BMI and WBC in the periodontitis group were significantly higher than the healthy group (Table 1). WBC was also more elevated for the periodontitis group in Chang et al. (2019). No correlation was found between the WBC numbers and the amount of growth factors in both groups (Chang et al., 2019). Various studies have shown that patients with higher BMI or chronic periodontitis tend to have higher WBC counts (Al‐Rasheed, 2012; Furuncuoglu et al., 2016; Kumar et al., 2014; Zekonis et al., 2014). The current study did not show any statistical differences in cell proliferation between healthy and periodontitis groups. Still, there was a trend for increased cell proliferation in the periodontitis group with HGF + PRF (Figure 2). The reason for this could be due to higher WBC levels (Al‐Rasheed, 2012; Furuncuoglu et al., 2016; Kumar et al., 2014; Zekonis et al., 2014) and more than 50% of the leucocytes being reported to be entrapped in a PRF clot (Dohan Ehrenfest et al., 2010) and thus supplementing cell growth.

In the current study, the PRF clot was compressed to form a PRF membrane, which is the most common form used in clinical dentistry. The results of the present study concerning HGF proliferation agreed with Vahabi et al. (2015). They showed that PRF membranes collected from a single healthy donor significantly induced HGF proliferation at 24 h (21 ± 1.73%, *p* ≤ 0.001) but in their study a negative proliferative trend was noticed at 48 and 72 h. The current study measured cell proliferation for 18 subjects (nine each for the healthy and periodontitis groups) at 72 h, and a wide range of cell responses was observed (Table 2 and Figure 1). These wide differences in results may be due to the limited number of subjects and could also be influenced due to individual variations between subjects. Another study (Fujioka‐Kobayashi et al., 2017) used PRF clots and found significant HGF proliferation at 1, 3, and 5 days with maximum cell proliferation at 5 days. The results of Fujioka‐Kobayashi et al. (2017) differed from Vahabi et al. (2015) and the current study due to the differences in the PRF processing. Our study found that PRF exudates induced significant cell proliferation at 3 days (Tables 3 and 4). Fujioka‐Kobayashi et al. (2017) used the entire PRF clot without any compression, and their results could be due to the combined effect of the PRF exudate and membrane toward cell proliferation. No other studies were found that evaluated the effects of PRF exudates on HGF and HPLF.

In conclusion, given the limitations of this study, this appears to be the first study to evaluate the role of both PRF exudates and membranes on HGF and HPLF proliferation. With the positive responses exhibited by the cells with PRF exudates irrespective of the subject's periodontal status, further studies are warranted to more closely examine the constituents of the PRF exudates and their role in cellular responses. The current study also evaluated HPLFs, and very similar trends in cell proliferation were noticed similar to HGFs (Table 5 and Figure 2). The HPLFs did not show as much proliferation as HGFs which might be due to their deeper location within the periodontium or their osteoblastic cell‐like nature (Jonsson et al., 2011). More studies with HPLF will help to understand the nature of these cells better. Due to the wide range of cell proliferation in different groups, studies with larger sample sizes may help develop more definitive answers. It is also vital to understand the role of other chronic systemic diseases (i.e., diabetes mellitus) or smoking status on the PRF quality. As there were no significant differences in cell proliferation from the PRF membranes or exudates between healthy and periodontitis subjects, one can conclude that PRF membranes and PRF exudates can be utilized as a potential material aid in wound healing and repair not affected by the periodontal status of the patients.

## Data Availability

The data that support the findings of this study are available from the corresponding author upon reasonable request.
